# Genetic basis of speciation and adaptation: from loci to causative mutations

**DOI:** 10.1098/rstb.2020.0503

**Published:** 2022-07-18

**Authors:** Jun Kitano, Asano Ishikawa, Mark Ravinet, Virginie Courtier-Orgogozo

**Affiliations:** ^1^ Ecological Genetics Laboratory, National Institute of Genetics, Yata 1111, Mishima, Shizuoka 411-8540, Japan; ^2^ Laboratory of Molecular Ecological Genetics, Graduate School of Frontier Sciences, The University of Tokyo, Kashiwanoha 5-1-5, Chiba 277-8562, Japan; ^3^ School of Life Sciences, University of Nottingham, Nottingham NG7 2RD, UK; ^4^ Institut Jacques Monod, CNRS UMR7592, Université Paris Cité, 15 rue Hélène Brion, 75013 Paris, France

**Keywords:** pleiotropy, convergent evolution, GWAS, QTL, genome editing, CRISPR/Cas

## Abstract

Does evolution proceed in small steps or large leaps? How repeatable is evolution? How constrained is the evolutionary process? Answering these long-standing questions in evolutionary biology is indispensable for both understanding how extant biodiversity has evolved and predicting how organisms and ecosystems will respond to changing environments in the future. Understanding the genetic basis of phenotypic diversification and speciation in natural populations is key to properly answering these questions. The leap forward in genome sequencing technologies has made it increasingly easier to not only investigate the genetic architecture but also identify the variant sites underlying adaptation and speciation in natural populations. Furthermore, recent advances in genome editing technologies are making it possible to investigate the functions of each candidate gene in organisms from natural populations. In this article, we discuss how these recent technological advances enable the analysis of causative genes and mutations and how such analysis can help answer long-standing evolutionary biology questions.

This article is part of the theme issue ‘Genetic basis of adaptation and speciation: from loci to causative mutations’.

## 1. Introduction

One of the fundamental goals of evolutionary biology is to understand how organisms and ecosystems have evolved in the past and will respond to changing environments in the future. To this end, we need to know whether evolution proceeds in small steps [1,2] or large leaps [3,4], how repeatable evolution is [5–7], and how constrained the evolutionary process is [8,9]. Understanding the genetic basis of phenotypic diversification and speciation in natural populations is key to properly answering these questions (e.g. [7,10]). Although most biologists would agree with the importance of genetic studies to decipher the evolution of living beings, their opinions sometimes differ regarding the amount of effort that should be dedicated to search for these genetic loci [11,12].

Genetic basis can be investigated at several different levels (table 1). First, using quantitative genetics approaches, we can investigate heritabilities, reaction norms (i.e. the range of phenotypes produced by a genotype, depending on the environment) and genetic correlations between phenotypic traits [13,14]. These quantitative genetics parameters help to predict how rapidly phenotypic traits would respond to selection and how evolutionary trajectories can potentially be biased by selection on other genetically correlated traits [13,15,16].

**Table 1.** Genetic basis for phenotypic variation in natural populations.

type of information	examples of data	examples of methods used
quantitative genetics parameters	heritability, gene × environment interaction (e.g. reaction norms), genetic correlations (e.g. *G*-matrix)	common garden experiment, parent–offspring regression, analysis of covariance between relatives
genetic architecture	location, linkage, number and effects of causative loci	quantitative trait loci (QTL) mapping, genome-wide association study (GWAS)
molecular mechanisms	types of causative genes, types and effects of causative mutations	genetic manipulations (e.g. transgenics and genome editing)

Second, the genetic architecture, such as the numbers, effect sizes, pleiotropy, genomic locations, epistasis and linkage of responsible loci for phenotypic variation, can be investigated using quantitative trait loci (QTL) mapping and genome-wide association studies (GWAS) [17–19]. In QTL mapping, crosses are performed between individuals varying for the trait of interest, and in the progeny population loci associated with phenotypic variation are identified. In GWAS, a natural population with genetic and phenotypic variation is used for identifying genetic loci associated with phenotypic variation within that population. Theoretical studies have demonstrated that information on the underlying genetic architecture can increase the predictability of the speed and reversibility of phenotypic evolution [17].

Finally, when responsible loci have large phenotypic effects, it is sometimes possible to identify the exact genes and mutations causing phenotypic variation and to investigate their functions at the molecular level [20–22]. Here, we first discuss how the investigation of causative genes and mutations at molecular levels will help answer long-standing questions in evolutionary biology. We note discussion of this topic has been controversial over the past two decades [23–26]. However, here we review new technological advances that increase the feasibility of identifying causative genes and mutations for adaptation and speciation.

## 2. What questions can be answered by identifying causative genes and mutations?

### (a) Does evolution proceed in small steps or large leaps?

Information about causative mutations can help to answer whether evolution proceeds in small steps [2,27] or large leaps [3,4], one of the long-standing questions in evolutionary biology [28,29]. QTL analyses have revealed the distributions of effect sizes of causative loci for many traits, and the distributions often follow the pattern of a small number of large-effect loci and a large number of small-effect loci [19,30–33]. Importantly, however, a single causative locus is not equal to a single causative mutation. There are many cases where a single causative locus is a composite of many linked mutations, and each mutation explains only a small fraction of the phenotypic variance [17,20,24,34]. For example, whereas the *bab* locus explains over 60% of the phenotypic variance of pigmentation between *Drosophila melanogaster* strains, each single nucleotide polymorphism (SNP) explains only a tiny fraction of the variance [35], suggesting that multiple mutations underlie this locus. This example clearly demonstrates that the effect sizes of QTL do not necessarily reflect the effect sizes of causative mutations. Furthermore, because QTL mapping can detect only large-effect loci, identified QTLs are typically biased toward large-effect loci [11,18,36]. Thus, in most cases QTL analyses or GWAS are not sufficient to understand the exact distribution of effect sizes of causative mutations.

For predicting very short-term evolution, it is often assumed that evolution proceeds via only pre-existing alleles without any new mutations. By an allele, we mean a linked genomic region that can contain multiple mutations. However, even for predicting short-term evolution, information on causative mutations and the distributions of their effect sizes is essential. For example, a large population is expected to have substantial input of mutations every generation because the number of mutations per generation in a diploid population with the population size of *N* would be theoretically 2*Nμ*, where *μ* is the mutation rate per generation [37]. Particularly in the case that mutations occur at mutational hotspots (see below), where mutation rates are higher than the genomic background by several orders of magnitude, new mutations cannot be ignored even for predicting short-term evolution [20,38–41]. Without a clear understanding of the identity of causative mutations and their effect size distribution, predictions of how evolution will proceed become inaccurate regardless of the timescale of evolution (i.e. short- versus long-term).

### (b) Is evolution repeatable?

Identification of causative mutations also contributes to a better understanding of the repeatability of evolution [7,42]. Genetic studies of convergent evolution have revealed hotspot genes that are repeatedly used for getting the same phenotypes [20,43–45]. In several cases, the same mutation repeatedly occurs at the same site in independent lineages [20,46,47]. For example, genomic analysis of microbes in experimental evolution has revealed the repeatability of mutations for adapting to the same environments [48,49]. In other cases, mutations occur at different sites in the same gene. For example, mutations at different amino acid sites in the *Mc1r* gene repeatedly caused adaptive pigmentation changes across a diverse array of animals [50].

There are several explanations for the presence of such hotspot genes. First, the number of available genes and mutations for achieving the same phenotype may be limited [47,51,52]. Second, mutations at hotspot genes may have optimal pleiotropy, with few detrimental effects associated with the diverse phenotypic traits affected by the mutation [43,53–55]. Pleiotropy can both facilitate and constrain adaptive evolution [2,28,54]. If a mutation changes a suite of multiple phenotypic traits in favourable directions, such a mutation with optimal pleiotropy is likely to be repeatedly used for adaptation to similar environments. Conversely, if a mutation changes only a certain phenotypic trait in a favourable direction but other traits in detrimental directions, such a mutation would be less likely to be frequently selected. By identifying the causative mutations and investigating their functional effects, it becomes possible to know whether the pleiotropic effect is due to the linkage of multiple mutations or multiple functions exhibited by a single mutation.

Third, hotspot genes may be located at genomic regions with high mutation rates [41,56–58]. Identification of causative mutations can also tell us whether convergent evolution is caused by mutations at mutational hotspots [38,39]. One of the examples showing the role of mutational bias in convergent evolution is the *Pitx1* gene, which underlies pelvic reduction of freshwater threespine stickleback populations [41]. A regulatory region of the *Pitx1* gene has a TG-nucleotide repeat with a fragile non-B DNA structure, and the repeated deletion of this regulatory region has been observed in multiple freshwater populations [41]. This example demonstrates that identifying causative mutations helps to understand whether mutational bias influences convergent evolution.

### (c) How constrained is the evolutionary process?

Identification of causative mutations can also deepen our understanding of the role of constraints in evolution. As described above, pleiotropy is one of the major constraints of adaptive evolution [2,16,59,60]. It is hypothesized that different types of mutations have different levels of pleiotropy [61]. *Cis*-regulatory mutations at enhancer regions enable tissue- or ontogenetic stage-specific modifications of gene expression and thus may have relatively low pleiotropic effects [43,62]. By contrast, amino acid changes and copy number variations can have larger pleiotropic effects because these changes can affect gene functions in multiple tissues throughout different ontogenetic stages. Importantly, these three types of mutations can occur together: there are many examples of duplicated genes diverging in both functional amino acid sequences and expression patterns [63,64]. We can directly test the difference in pleiotropic effects between different types of mutations by functional analysis of each type of mutation.

Epistasis, an interaction between a mutation at one genomic site and a mutation at another site, can make particular combinations of mutations more favourable than others, thus constraining the evolutionary trajectories [65–67]. Except for several cases of experimental evolution *in vitro* [68], epistatic effects have been generally investigated between loci but not between mutations. Using large-scale GWAS, it would be possible to investigate epistasis between mutations for fitness (Villoutreix *et al.* [69] in this issue). Alternatively, epistasis among mutations can be investigated using *in vivo* genetic engineering, as nicely demonstrated in the example of the *shavenbaby* locus contributing to variations in larval cuticular patterns between *Drosophila* species [70].

### (d) Other benefits of identifying causative genes and mutations

Once we identify causative genes and mutations, we can try to infer when the adaptive mutations arise, how they spread in a population, and what their fitness effects are. For example, genomic sequence data can help to infer the age of an adaptive mutation, selection regimes and the past allele frequency trajectories [71–73]. This approach will tell us whether a particular mutation occurred before or after environmental changes or the completion of speciation [74]. Furthermore, using semi-natural field experiments, we can investigate the fitness effects of particular alleles and mutations [75].

It is also important to identify the exact mutations not only for basic science but also for various applications [76]. To raise a few examples, we can transfer pathogen-resistance genes to tomatoes for agriculture [77]. By looking for specific mutations in aquatic organisms, we can test for the previous presence of a pollutant in their habitats [78]. By counting the copy number of key adaptive genes for freshwater survival, we may be able to predict which fishes are amenable to freshwater aquaculture ([79] and Ishikawa *et al*. [80] in this issue).

Identification of causative genes and mutations would also help to understand the link between organismic evolution and ecological processes. Organismic evolution can influence not only the population dynamics but also the community structures [81]. As reviewed by Yamamichi [82] in this issue, the genetic architecture underlying an adaptive trait in an organism can influence several ecological processes. However, little is known about how much a single mutation or allele can influence the ecological community in nature and how prevalent genes with disproportionately large effects on the ecological processes, called keystone genes, are [83,84].

## 3. How to go beyond candidate loci to identify causative genes and mutations in natural populations

Recent advances in genome sequencing technologies have made it increasingly easier to identify candidate loci associated with adaptation and speciation. For example, QTL mapping and GWAS have identified many candidate loci associated with phenotypic variation and reproductive isolation in natural populations (e.g. Brien *et al.* [85], Gloss *et al.* [86] and Peter *et al.* [87] in this issue). Although large-scale GWAS enables the investigation of the genetic architecture at a far higher resolution than quantitative trait loci (QTL) mapping [69,86,87], linkage disequilibrium within a causative locus often precludes us from pinpointing causative genes and mutations solely by GWAS. For example, chromosomal inversions can capture adaptive linked mutations (e.g. Maney & Küpper [88] and Villoutreix *et al*. [69] in this issue). So how can we go beyond simply identifying causative loci and move toward investigating causative genes and mutations?

Detailed *in vivo* functional analysis of each mutation is a powerful way to investigate the effect of each mutation, as demonstrated by several excellent studies. For example, polymorphism at the *adh* gene is responsible for a 2.5–3- fold difference in the enzymatic activity of alcohol dehydrogenase between *Drosophila melanogaster* strains. However, this major locus contains multiple mutations at the intron, protein-coding region and 3′-untranslated region of the *adh* gene [89]. *In vivo* genetic engineering showed that different sites have different effects on enzymatic activity and also show epistatic interactions [89]. Using systematic synthetic biology approaches (e.g. Crocker *et al.* [90] in this issue), it is now possible to artificially generate multiple genotypes that do not exist in natural populations and to investigate the evolvability and constraint of evolution.

Until recently, genetic manipulation has largely been limited to laboratory model organisms. Functional validation of candidate mutations was conducted mainly using laboratory model organisms, such as *Saccharomyces cerevisiae*, *Arabidopsis thaliana* and *Drosophila melanogaster* ([91–93] and Tsuchimatsu & Fujii [94] in this issue). Even when genetic changes are identified in non-laboratory models, such as deer mice, voles and cichlid fishes, the mutational effects have been examined in laboratory model organisms, such as the mouse and the zebrafish [95–97].

Technological advances in genome editing using CRISPR/Cas systems and site-specific recombinases are now making it possible to conduct functional assays of candidate genes directly in so-called non-model organisms [98–101]. CRISPR/Cas systems have been recently applied to functional analysis of genes underlying naturally occurring phenotypic variation in natural populations, such as Sulawesian medaka fishes [102] and *Heliconius* butterflies [103]. CRISPR/Cas systems have been most frequently used for gene knockout, i.e. the generation of loss-of-function mutations, which enables us to investigate the function of candidate genes. Recent technological advances in CRISPR/Cas systems are making it possible to induce nucleotide substitutions of interest in a target gene (a technique named ‘allele replacement’) and chromosomal rearrangements [104,105], as reviewed by Ansai and Kitano [104] in this issue. Although these genome editing technologies often have off-target effects [106], we should eventually be able to remove such undesirable mutations by backcrossing or future technological advances.

## 4. Conclusion

Although hundreds of genes important for development and physiology have been identified in laboratory model organisms during the last 50 years, we still do not know much about the molecular changes and causative mutations underlying naturally occurring phenotypic variation that is important for adaptation and speciation in natural populations. With the advance of genome sequencing and genome editing technologies, we are now in a position to identify many causative genes and mutations in natural populations of model and non-model species alike and to begin to tackle several long-standing evolutionary questions. We hope that the collection of papers in this theme issue will encourage research in that direction.

## Data Availability

This article has no additional data.
